# Clinical Note—Folic Acid
*Summarized from J. F. Wilkinson, *B.M.J.,* 1948, i, 771. Ib. 822; *Lancet,* editorial, 1948, i, 371; Davidson and Girdwood, *Lancet,* 1948.


**Published:** 1948

**Authors:** 


### CLINICAL NOTE—FOLIC ACID *

Folic acid, a member of the vitamin B group, was first isolated from spinach. Similar substances were later extracted from various sources: yeast, liver, kidney, milk, mushrooms, green leaves, etc. In 1945 pteroylglutamic acid was synthesized, and to judge by the effects of its administration appears to be identical with folic acid. The latter is necessary for the growth of lactobacillus casei and of streptococcus faecalis: this property forms the basis of methods of estimation.

*Pernicious Anaemia.* The immediate effects of treatment with adequate doses of folic acid, oral or by injection, are good, though not as good as an active liver or stomach extract. In most cases 20 mgm. per diem orally will produce clinical remission and the maintenance dose is 5–15 mgm. But even when the blood count has become normal there is a tendency for macrocytes to persist. Unfortunately however neurological symptoms do not resolve. Out of 184 cases treated, in fifty-nine neurological symptoms appeared or became worse during treatment. Four of Wilkinson's patients, originally free from nerve changes, developed subacute combined degeneration of the cord within two months and during treatment. Others have recorded similar experiences.

The changes which occur in the cord during treatment with folic acid differ from those occurring naturally in the course of the disease. The onset is more acute and when liver extract is given recovery is much more rapid. From this it has been inferred that folic acid interferes in some way with the nutrition of the cord. So far subacute combined degeneration of the cord during treatment of other diseases with folic acid has not been reported. But Davidson and Girdwood, treating steatorrhoea, record two cases of severe peripheral neuritis which cleared up when liver extract was injected. They suggest that disproportion in the constituents of the vitamin B group may be responsible for the nerve changes. There is general agreement that folic acid has no place in the treatment of pernicious anaemia.

*Idiopathic steatorrhoea* and *Coeliac disease.* In these conditions a variable result is obtained. Patients with megaloblastic marrows often show considerable improvement in the blood picture for a time, but tend to relapse. In those with normoblastic marrows the results are not so good: there is usually some clinical improvement and diarrhoea is less but fat absorption remains defective.

Even when massive doses are given there is no improvement in refractory macrocytic anaemia, aplastic anaemia, agranulocytosis, neutropenia, thrombocytopenia, leukaemia, or chronic ulcerative colitis.

* Summarized from J. F. Wilkinson, *B.M.J.*, 1948, i, 771. Ib. 822; *Lancet*, editorial, 1948, i, 371; Davidson and Girdwood, *Lancet*, 1948.

